# Validation of an improved questionnaire assessing the social cognitive constructs of the Health Action Process Approach among parents regarding brushing their children’s teeth

**DOI:** 10.1371/journal.pone.0300432

**Published:** 2024-06-04

**Authors:** Karin Alexandra van Nes, Cor van Loveren, Irene Helena Adriana Aartman

**Affiliations:** 1 Department of Paediatric Dentistry, Academic Centre for Dentistry, University of Amsterdam and VU Amsterdam, Amsterdam, The Netherlands; 2 Department of Oral Public Health, Academic Centre for Dentistry, University of Amsterdam and VU Amsterdam, Amsterdam, The Netherlands; Shahid Beheshti University of Medical Sciences School of Dentistry, ISLAMIC REPUBLIC OF IRAN

## Abstract

**Objective:**

The Health Action Process Approach (HAPA) describes social cognitive constructs related to behaviour change. A validated questionnaire is needed to measure these constructs in paediatric dentistry. The aim of this study was to improve an existing HAPA-based questionnaire for parents regarding brushing their children’s teeth and to assess its validity and reliability in a population of parents of high caries risk children.

**Methods:**

Parents of high caries risk children of 3–10 years filled out the adjusted HAPA-based questionnaire. Mokken scale analysis, graded response model analyses, factor analyses and reliability analyses were performed according to the protocol of Dima. Discriminant validity was assessed by comparing the mean scores of the HAPA constructs between two groups of participants, based on different levels of caries experience, brushing frequency and education level of the mother.

**Results:**

The Mokken scale analysis and factor analyses indicated a multidimensional eight factor scale. The graded response model did not fit our data. The subscale action control could be identified as a two-factor subscale. Reliability indices from the Dima protocol varied, for instance Cronbach alpha ranged from 0.73 to 0.96. The constructs coping self-efficacy, action planning and action control discriminated between brushing frequencies.

**Conclusions:**

The adjusted HAPA-based questionnaire is an improved, valid and reliable instrument that could be used to evaluate HAPA-based interventions to improve children’s oral health.

## Introduction

Improving health behaviour is one of the greatest challenges in preventive dentistry. In order to take on these challenges, social cognitive constructs that are related to health behaviour change have been identified and modelled into behaviour change pathways [1]. The Health Action Process Approach (HAPA) is a health behaviour change model that aims to bridge the gap between motivation and behaviour by facilitating the conversion of intention to actual behaviour [2]). Schwarzer and Hamilton [3] define this approach as ‘a general framework to conceptualise health self-regulation as a process that can be divided into phases with constructs’. A motivational phase and a volitional phase are two stages a person goes through during the behaviour change process. Each phase contains specific social cognitive constructs. First, in the motivational phase a person forms the intention to perform new behaviour based on the social cognitive constructs risk perceptions, outcome expectancies, action self-efficacy and intention. Subsequently, in the volitional phase a person plans the new behaviour and how to cope with potential barriers with help of the social cognitive constructs action planning, coping planning, coping self-efficacy and action control (S1 Fig and S1 Table). HAPA has been successfully applied in numerous settings, such as physical activity, seatbelt use, hand hygiene, quitting smoking and dietary behaviour [4, 5]. In oral health the HAPA has been used for dental flossing [6, 7] and tooth brushing [8–10].

Gholami and Schwarzer [11] have provided HAPA questionnaires to measure the social cognitive constructs of the HAPA model for a variety of health behaviours. Alternative versions of these questionnaires have subsequently been constructed and validated for specific health behaviours in specific groups, such as physical activity among schizophrenia patients [12], physical activity among diabetic patients [13, 14], treatment adherence among haemodialysis patients [15] and condom use among high school children [16]. A variety of validation methods have been used in these studies; all of them used factor analysis to identify latent variables to represent the social cognitive constructs and Cronbach alphas to measure reliability. Additionally, some researchers performed structural equation modelling using confirmatory fit analysis and path analysis to confirm the model [13, 16]. While the type of validation analysis varied, all the researchers concluded that the questionnaires could be used successfully in HAPA-based interventions. However, in oral health studies, the validation processes used for the HAPA-questionnaires have not been reported [11].

The HAPA model could be a promising model to facilitate parents’ oral hygiene behaviour for their children. Young children depend mostly on their parents for oral healthcare, such as tooth brushing. Therefore, an intervention to change the behaviour in this target group should focus on the parents. Hamilton et al. (8) have determined social cognitive constructs in parents regarding supervised tooth brushing to investigate the mediation effects of some of the constructs in the HAPA model. They showed that self-efficacy, planning and action control were mediators that bridged the gap between intention and actually performing the supervised tooth brushing.

To determine the HAPA constructs in parents regarding brushing their children’s teeth, a valid and reliable questionnaire is needed. Previously, a questionnaire has been developed for this purpose [17], but validity and reliability analyses showed that the questionnaire was mostly a unidimensional scale and the HAPA constructs could not be properly identified. Thus, considerable revision of the questionnaire was required. The aim of the current study was to improve the previously developed HAPA-based questionnaire for parents regarding brushing their children’s teeth in a population of parents of high caries risk children by identifying the individual constructs and to assess its validity and reliability.

## Materials and methods

### Ethical statement

The study was part of a more extensive study, the protocol of which was approved by the medical ethical committee of the VU University as non-Medical Research Involving Human Subject Act, protocol number 2018–021. The consent procedure implied that the parents were informed verbally and in writing about the research, and signed the informed consent form before the study commenced.

### Participants

In this study parents/caregivers (hereafter referred to as ‘parents’ as 98% of them were the actual parents) of high caries risk children referred for the treatment of caries to a paediatric dental referral practice in the Netherlands were approached to participate from May 2018 until April 2020. In this practice, dental rehabilitations were carried out either in multiple treatment session after habituation sessions in which the paediatric dentist taught the children coping skills, or in one session under intravenous sedation. The parents were included in the study when their children were healthy (ASA I) [18], aged 3–10 years, with at least one cavity in at least three quadrants of the dentition, when they had sufficient understanding of the Dutch language to fill out a HAPA-based questionnaire and after they had signed the informed consent. Parents were excluded when their children had enamel abnormalities other than caries or syndromic abnormalities of the teeth. The parents were allowed to participate for one child only.

At intake, a research assistant informed the parents verbally about the study procedure and provided them with written information thereof. They were invited to participate and were assured that they could withdraw from the study at any time without negative consequences. If the parents agreed to participate, they were invited to fill out the questionnaire at intake. The paediatric dentist recorded the number of teeth (t/T, lowercase for the primary dentition and uppercase for the permanent dentition) that were decayed (d/D), missing due to caries (m/M), or filled (f/F) as ‘dmft+DMFT’ on a registration form at intake. A tooth was considered ‘decayed’ when caries had clinically progressed into the dentine.

From May 2018 to April 2020, 176 parents were willing to participate and met the inclusion criteria. Of these, 163 parents filled out 165 questionnaires. Two questionnaires were removed because two parents filled out questionnaires for two children, one questionnaire was removed due to more than 13 missing values on the HAPA items, as well as two questionnaires that were considered to be outliers, as will be described below. Finally, a total of 160 questionnaires was used for the statistical analysis (https://figshare.com/s/37ef5ace3afd6893143a). The demographic characteristics of the sample are shown in Table 1. The data collection was part of a longitudinal study on changes in the HAPA subscales in parents of high caries risk children who were treated in a paediatric dental practice. For the current study, only data collected at intake were used.

**Table 1:** Characteristics of the total sample (*n* =160).

Variable	Categories	*N*	Perc.
Child’s gender	Male	87	54.4
	Female	73	45.6
Child’s country of birth	The Netherlands	149	93.2
	European Union (The Netherlands excluded)	2	1.2
	Other	6	3.6
	NA	3	1.9
Mother’s educational level	Low	99	61.9
	High	51	31.9
	NA	10	6.3
Accompanying person’s relation to child	Mother	118	73.8
	Father	35	21.9
	Mother and father	4	2.5
	Other	1	0.6
	NA	2	1.3
Accompanying person’s marital status	With partner	126	78.8
	Single	27	16.9
	NA	7	4.4
Variable	*Mean*	*SD*	Range
Child’s age (n = 158)	5.2	1.5	3–9
Mother’s age (n = 157)	34.2	5.6	22–48
dmft+DMFT (n = 160)	8.4	2.4	3–14
Brushing frequency* (n = 157)	1.8	0.5	0–3.5

Note

Perc. = percentage

NA = not available: missing values

*open-ended question ‘*In the past week*, *how many times* *a day* *have you brushed your child’s teeth*?*’*

### HAPA questionnaire

A previously-developed HAPA questionnaire was adjusted according to the suggestions made after its validation [17]. This questionnaire, in Dutch, consisted of items designed to assess the HAPA subscales, as well as the following demographic and oral health variables: gender, age of the child, country of birth, age of the mother, relationship to the child, marital status, caries experience and an open-ended item on brushing frequency, as well as an item on brushing frequency on an ordinal scale.

Based on the results of the previous study [17], we made adjustments to the questionnaire. Firstly, the open-ended question on brushing frequency was reformulated from “*How many times a day in the past week did you brush your child’s teeth*?”into ‘*In the past week*, *how many times* *a day* *have you brushed your child’s teeth*?’ Secondly, one unscalable item of the subscale risk perceptions was removed, namely ‘*If I don’t brush my child’s teeth daily then my child will need braces in the future*’. Thirdly, because the original items of the intention subscale were perceived as being contradictory, two additional items were added to the questionnaire. These were: *‘In the following period I intend to brush the teeth of my child myself’ and ‘In the following period*, *I intend to check the teeth of my child after brushing’*. Fourthly, the double-barrelled item of action planning was split into two items, namely ‘*I have made a concrete plan where to brush my child’s teeth*’ and *‘I have made a concrete plan when to brush my child’s teeth’*. Lastly, to form a subscale that represented action control more comprehensively, two items were removed because they did not represent the construct properly. These items were ‘ *During the past week often I had my intention of brushing my child’s teeth on my mind*’ and ‘ ‘*During the past week I really tried to brush my child’s teeth daily’*. Also, five new items were added. Those were ‘*In the past week I knew exactly when I skipped brushing my child’s teeth’;* ‘*In the past week I have kept track of what prevented me from brushing my child’s teeth’;* ‘*In the past week my child carefully kept track of how often I brushed his/her teeth*’; ‘*In the past week I have been very involved in brushing my child’s teeth’;* and ‘*In the past week I really tried to reach the goals that I have set for brushing my child’s teeth’*. The final version of the questionnaire consisted of 35 HAPA-based items. Of these 35 HAPA items, one item was an open-ended question to measure ‘behaviour’, namely ‘*In the past week*, *how many times* *a day* *have you brushed your child’s teeth*?’. The other 34 HAPA items could be answered on a Likert scale to measure the eight HAPA subscales (Table 2). The Likert scale ranged from *absolutely not true* (1) to *not true* (2), *true* (3) and *absolutely true* (4) for the HAPA items of the subscales intention, action self*-*efficacy, coping self*-*efficacy, action planning, coping planning and action control, and from *most unlikely* (1) to *unlikely* (2), *likely* (3) and *most likely* (4) for the subscales outcome expectancies and risk perceptions. The mean score of the items was calculated for each subscale and a maximum of one missing value per subscale was allowed. Higher scores indicated a more positive cognition. Then, the items were grouped per construct using section breaks and highlighting item stems, whereas the items were inter-mixed in the preceding study.

**Table 2:** HAPA items and their response frequencies*, mean score and standard deviations.

Subscale	item	Subscale item stem and item	*N*	Item-frequency distribution				*Mean score*	*SD*
				1	2	3	4		
Outcome expectancies (OE)		If I brush my child’s teeth on a daily basis…		most unlikely	unlikely	likely	most likely		
	OE1	…people in my community will see that my child is a clean person	155	21	35	75	23	2.65	0.89
	OE2	. . .my child will remain having healthy teeth	156	3	7	84	58	3.28	0.65
	OE3	. . .my child will feel good with beautiful teeth	155	3	5	83	62	3.33	0.64
Risk perceptions (RP)		If I don’t brush my child’s teeth daily then…							
	RP1	. . .my child will be at risk for developing gum diseases	159	3	10	85	60	3.27	0.67
	RP2	. . .my child will be at risk for developing tooth decay	159	1	3	72	83	3.49	0.57
	RP3	. . .then the new permanent teeth will be harmed	159	2	7	68	81	3.44	0.65
	RP4	. . .then my child might lose his/her teeth too soon	159	0	13	63	80	3.42	0.65
	RP5	. . .then my child might have bad breath	159	1	9	64	83	3.45	0.64
Action self-efficacy (aSE)		I am confident that I immediately can start brushing my child’s teeth daily…		absolutely not true	not true	true	absolutely ture		
	aSE1	. . .even if I have to force myself to do so	158	5	4	70	78	3.40	0.70
	aSE2	. . .even if it is time consuming	158	4	6	68	80	3.42	0.69
	aSE3	. . .even if others do not brush their children’s teeth	159	6	5	60	88	3.45	0.74
Intention (INT)		In the period ahead, I intend to….							
	INT1	… to brush my child’s teeth properly once a day	155	19	30	54	52	2.91	1.00
	INT2	. . . brush my child’s teeth myself	151	4	14	78	52	3.20	0.72
	INT3	… check my child’s teeth after brushing	151	3	3	80	63	3.36	0.62
	INT4	… brush my child’s teeth properly at least twice a day	158	1	9	61	84	3.46	0.64
Coping self-efficacy (cSE)		I am confident that I can continue daily brushing my child’s teeth…							
	cSE1	. . .even when I cannot see any positive changes immediately	158	0	1	77	80	3.50	0.51
	cSE2	. . .even when my child does not cooperate	159	0	5	76	77	3.45	0.56
	cSE3	. . .even when I am in a hurry	159	0	5	81	73	3.43	0.56
	cSE4	. . .even when it takes a long time to become part of my routine	159	0	1	77	81	3.50	0.51
Action planning (AP)		I have made a concrete plan…							
	AP1	….where to brush my child’s teeth	152	0	21	88	43	3.14	0.64
	AP2	….when to brush my child’s teeth	153	0	5	94	54	3.32	0.53
	AP3	. . .how often to brush my child’s teeth	157	0	4	89	64	3.38	0.54
	AP4	. . .how to brush my child’s teeth	156	0	9	94	53	3.28	0.57
	AP5	. . .how much time to spend with brushing my child’s teeth	157	0	15	90	52	3.24	0.61
Coping planning (CP)		To keep brushing my child’s teeth in difficult situations, I have made a concrete plan…							
	CP1	…in case something interferes with brushing my child’s teeth	154	3	39	78	33	2.92	0.74
	CP2	. . .in case I am in a hurry	154	3	42	73	35	2.91	0.76
	CP3	. . .in case my child does not cooperate	154	4	37	77	35	2.94	0.74
	CP4	…in case my child has pain. bleedings gums or tooth decay	156	5	35	76	39	2.96	0.78
Action control (AC)		In the past week…							
	AC1	…I have consistently monitored how. when and how often I have brushed my child’s teeth	159	9	52	71	27	2.73	0.81
	AC2	… I knew exactly when I skipped brushing my child’s teeth’	149	9	35	80	24	2.81	0.78
	AC3	… I have kept track of what prevented me from brushing my child’s teeth’,	146	15	66	55	10	2.41	0.77
	AC4	… **my child** carefully kept track of how often I brushed his/her teeth	151	23	63	47	15	2.37	0.86
	AC5	. . .I have been very involved in brushing my child’s teeth	153	5	33	84	29	2.90	0.73
	AC6	. . . I really tried to reach the goals that I have set for brushing my child’s teeth’	152	4	22	85	40	3.06	0.72

Note

*without outliers, before missing data analyses.

### Statistical analyses

We performed scale analyses according to the Dima protocol [19]. These analyses included data control, [20], Mokken scale analysis, parametric item response theory analysis, factor analysis and reliability analysis. The total subscale scores and standard deviations were computed based on these analyses. A detailed description can be found in Van Nes et al. [17]. To verify the results of the Dima protocol, we performed principal component analysis (PCA). Additionally, we compared whether constructs that are supposed to be related actually are related in our data. The constructs of the HAPA model are supposed to be related since they are indicative of the level of motivation and performance of parents to brush their children’s teeth. Furthermore, good oral health is a result of the performance of positive oral health behaviour, which is easier to adopt when you have high mean scores on the HAPA constructs. Therefore, we hypothesized that higher mean scores on the HAPA constructs are associated with higher mean brushing frequency.Thus, we calculated the Pearson correlation coefficients between the subscales to measure convergent validity. To measure the discriminant validity of the questionnaire, we analysed the relationships between the scores on the HAPA subscales and brushing frequency (the open-ended question ‘*In the past week*, *how many times* *a day* *have you brushed your child’s teeth*?’) using Pearson correlation coefficients.

The analyses were performed using IBM SPSS Statistics (Version 27) for missing value analysis (Little’s MCAR test [21]), Pearson correlations, independent sample *t*-tests and sample adequacy (Kaiser-Meyer-Olkin measure of sampling adequacy for principle component analysis [22]. In addition, the open source program R (Version 1.4.1717) [23] with the following packages was used for other analyses: mokken [24], psych [25], ltm [26], msm [27], polycor [28] and lavaan [29]. The R-code can be obtained from the corresponding author upon request. A significance level of 1% was used for all the statistical analyses.

## Results

### Dima protocol analyses

#### Step 1. Data control

No invalid data imputation was observed. The response frequencies varied sufficiently (Table 2). Missing value analysis showed that 151 missing values were missing completely at random (Little’s MCAR test: Chi-square 726.235, *df* = 767, *p* = 0.851). These items were replaced with plausible item scores using two-way imputation analysis [30]. Outlier detection for ordered rating scales data detected that two respondents had idiosyncratic response patterns and these outlying questionnaires were removed [20]. After outlier removal, eight non-significant negative Pearson correlations coefficients were observed between items (S2 Table). We judged that no reverse coding was necessary for any of the items and the items could, therefore, be retained in the data set

#### Step 2. Mokken scale analysis

The entire set of 34 items did not form a unidimensional scale (*H* = 0.357,*se* = 0.028) (Table 3). Several deviant items were identified with *automated item selection procedure*, while several violations were observed of *manifest monotonicity* and *local independence* (Table 3). Indicating that for the total scale the probability that a person endorsed an item was not based on the latent trait and the items were probably not only related with each other via the latent trait. Only the anticipated four-item subscale intention formed a ‘medium’ scale (*H* = 0.439, *se* = 0.066). All the other anticipated subscales could be labelled as ‘strong’ unidimensional scales *H*>0.5 [31]. The item scalability coefficients were acceptable (*Hi*> 0.3) for each anticipated subscale. Nevertheless, deviating but scalable coefficients were observed for item INT1 (*Hi* = 0.353, *se* = 0.078) and item INT4 (*Hi* = 0.345, *se* = 0.086) in the anticipated four-item subscale intention and for item AC2 in the anticipated six item subscale action control (*Hi* = 0.353, *se* = 0.094). Each item in each subscale discriminated well between persons. There were no significant violations of *manifest monotonicity* and no violations of *manifest invariant item ordering* in any subscale. Two item pairs were flagged for *local independence* [CP1-CP2 and CP2-CP4] [32]. The *H*^*T*^ ranged from -0.05 for action self-efficacy to 0.753 for outcome expectancies (Table 3). The item response function (IRF) plots of the subscales were not conclusive; thus, it was not clear whether the subscales met the assumption of the *double monotonicity* model, i.e., whether all subjects perceived the items in the same rank order of difficulty. Based on item scalability coefficients and item content, we decided to remove item INT1 from the subscale intention and item AC2 from the subscale action control in further analyses.

**Table 3:** Measures for the entire set of items Mokken scale analysis, parametric item response theory analysis.

Scale/ subscale	Item	Aisp Entire set of items	*H*_i_ (se) Entire set of items	*H*_*i*_ (se) subscale	*H* (se)	#vi MIIO Entire set of items	#vi LI Entire set of items	*H* ^*T*^	CITR Entire set of items	CITR Remaining set of items	CITR subscale
Entire set of items					0.357(0.028)						
OE											
	OE1	3	0.166 (0.065)	0.471 (0.089)	0.571 (0.72)			0.753	0.24	0.24	0.38
	OE2	3	0.244 (0.060)	0.614 (0.069)					0.39	0.40	0.62
	OE3	3	0.193 (0.063)	0.632 (0.072)			1		0.31	0.32	0.63
RP											
	RP1	1	0.287 (0.045)	0.708 (0.050)	0.704 (0.045)			0.136	0.45	0.46	0.75
	RP2	1	0.321 (0.047)	0.763 (0.039)					0.48	0.49	0.88
	RP3	2	0.271 (0.046)	0.748 (0.039)					0.44	0.45	0.85
	RP4	2	0.275 (0.048)	0.720 (0.041)					0.43	0.44	0.80
	RP5	2	0.249 (0.056)	0.589 (0.082)					0.39	0.40	0.66
aSE											
	aSE1	1	0.384 (0.044)	0.919 (0.047)	0.924 (0.036)		1	0.005-	0.62	0.62	0.90
	aSE2	1	0.408 (0.040)	0.946 (0.022)					0.66	0.66	0.95
	aSE3	1	0.408 (0.039)	0.907 (0.043)					0.64	0.64	0.86
INT											
	INT1	0	0.149 (0.056)	0.353 (0.078)	0.439 (0.066)	7(4)	1	0.295	0.23		0.43
	INT2	1	0.291 (0.054)	0.513 (0.066)					0.45		0.73
	INT3	1	0.342 (0.054)	0.574 (0.065)					0.52		0.78
	INT4	1	0.330 (0.054)	0.343 (0.086)					0.51		0.49
INT (3items)											
	INT2			0.561 (0.079)	0.571 (0.072)			0.175		0.42	0.65
	INT3			0.660 (0.062)						0.50	0.79
	INT4			0.493 (0.094)						0.50	0.54
cSE											
	cSE1	1	0.511 (0.035)	0.923 (0.025)	0.912 (0.021)			0.195	0.78	0.78	0.94
	cSE2	1	0.483 (0.034)	0.919 (0.023)					0.76	0.76	0.93
	cSE3	1	0.450 (0.036)	0.901 (0.028)					0.70	0.71	0.87
	cSE4	1	0.489 (0.036)	0.907 (0.029)					0.74	0.75	0.92
AP											
	AP1	1	0.474 (0.036)	0.838 (0.043)	0.866 (0.030)			0.335	0.71	0.71	0.80
	AP2	1	0.494 (0.036)	0.869 (0.032)					0.74	0.75	0.88
	AP3	1	0.501 (0.036)	0.920 (0.033)					0.77	0.78	0.90
	AP4	1	0.507 (0.032)	0.865 (0.034)					0.77	0.78	0.90
	AP5	1	0.493 (0.034)	0.843 (0.037)					0.76	0.77	0.87
CP											
	CP1	1	0.492 (0.036)	0.905 (0.025)	0.886 (0.026)	2(1)	1	0.023	0.70	0.70	0.95
	CP2	1	0.499 (0.034)	0.912 (0.024)		4(2)			0.73	0.72	0.95
	CP3	1	0.494 (0.034)	0.880 (0.026)		4(1)			0.71	0.71	0.92
	CP4	1	0.477 (0.034)	0.848 (0.035)		4(2)			0.70	0.70	0.85
AC											
	AC1	1	0.311 (0.052)	0.518 (0.067)	0.507 (0.060)			0.365	0.46		0.65
	AC2	1	0.267 (0.065)	0.353 (0.094)					0.39		0.42
	AC3	4	0.219 (0.060)	0.516 (0.069)			1		0.32		0.64
	AC4	4	0.246 (0.059)	0.550 (0.060)			1		0.36		0.71
	AC5	1	0.377 (0.054)	0.540 (0.066)		4(1)			0.56		0.74
	AC6	1	0.369 (0.055)	0.571 (0.060)		4(1)			0.55		0.75
AC (5items)											
					0.587 (0.057)						
	AC1			0.578 (0.073)				0.488		0.45	0.65
	AC3			0.560 (0.069)						0.31	0.65
	AC4			0.603 (0.060)						0.35	0.73
	AC5			0.594 (0.069)						0.58	0.73
	AC6			0.594 (0.062)			1			0.54	0.73

Note

Aisp = automated item selection procedure, with lower bound c = 0.3

*H*_*i =*_ item scalability coefficient

*H =* scalability coefficient

#vi = number of violations with the significancy between parenthesis

MIIO = manifest invariant item ordering

*H*^*T*^ = item ordering coefficient

LI = local independence

CITR = item-total correlation corrected for item overlap and scale reliability

#### Step 3. Parametric item response theory analysis

We combined response categories 1 and 2 to test the fit of the graded response model, since the items coping self-efficacy, action planning and item RP4 had no responses in the first response category. The graded response model with free parameters had the best fit, compared to graded response model with fixed parameters, for the remaining set of items and most of the subscales. For the subscales action planning and coping planning, however, the graded response model with fixed discrimination parameters fitted better. The lack of fit of the item pairs and item triplets suggested that the graded response models fitted neither the remaining set of items nor the subscales.

#### Step 4. Factor analysis

Parallel analysis for the remaining set of 32 items [INT1 and AC2 removed] suggested eight factors. The scree plots and the plots of the very simple structure analysis (VSS) suggested one main primary factor and five other factors for this remaining set of items. Plots of the parallel analysis and VSS of the subscales suggested two factors for the subscale action control only. One factor was suggested for the other subscales. A hierarchical item cluster analysis for the remaining set of items initially identified four separate clusters: action control, ‘risk perception’, coping planning and a fourth cluster. From this fourth cluster, a set of items split off as clusters in the following order: outcome expectancies, intention, action planning and action self-efficacy.

#### Confirmatory fit analysis

We fitted multiple models and used Hooper’s [33] benchmarks (Table 4). First, the remaining set of items did not fit a one-factor model. Then, the remaining set of items fitted the anticipated eight-factor model, with some measures slightly below the threshold. Furthermore, following the results of the preceding analyses, we divided the action control items into two factors (‘action control awareness’ [AC5 and AC6] and ‘action control monitoring’ [AC1, AC3 and AC4]) and fitted a nine-factor model, which fitted somewhat better (Table 4).

**Table 4:** Estimates of confirmatory fit analysis and their benchmarks(33).

Fit statistic	Model			Benchmark(33)
	1-Factor model (32 items)	8-Factor model (32 items)	9-Factor model (32 items)	
χ^2^(*df*)	2839*(464)	732.969* (436)	652,725* (428)	
RMSEA	0.179*	0.065 (*p* = 0.002)	0.057 *p* = 0.089)	<.06
TLI	0.427	0.924	0.941	≥.95
CFI	0.464	0.933	0.949	≥.95
SRMR	0.127	0.060	0.059	<.08
GFI	0.431	0.786	0.809	≥.95
AGFI	0.353	0.741	0.764	≥.95

Note

χ^2^ (*df*) = chi square and degrees of freedom

RMSEA = root mean square error of approximation

TLI = Tucker-Lewis index

CFI = Comparative Fit Index

SMRA = Standardized root mean square residual

GFI = Goodness-of-fit statistic

AGFI = Adjusted goodness-of-fit statistic

^*^p<0.001

#### Step 5. Reliability

The reliability for the total scale was excellent (Table 5). For the subscales, Cronbach’s alpha (α) varied from 0.68 to 0.96, Revelle’s beta (β) ranged from 0.54 to 0.92, McDonald’s omega hierarchical (ω_h_) ranged from 0.65 to 0.95 but were not calculated for intention, action self-efficacy and coping planning, and Guttman’s lambda-6 (λ_6_) ranged from 0.69 to 0.95 (Table 5).

**Table 5:** Reliability measures, mean scores and standard deviations after imputation of missing values for the remaining set of items and the subscales.

Scale	Reliability coefficient				*M*	*SD*	Discriminant validity			
									Brushing frequency a day	
	α	β	ωh	λ_6_					*r*	*p-*value
OE	0.73	0.54	*Nc*.	0.7	3.1	0.58			0.08	0.308
RP	0.91	0.8	0.86	0.9	3.4	0.56			0.09	0.259
aSE	0.95	0.92	0.95	0.95	3.4	0.67			0.13	0.108
INT^**a**^	0.73	0.63	*Nc*.	0.69	3.4	0.53			0.01	0.869
cSE	0.96	0.92	0.94	0.95	3.5	0.52			0.20	0.015
AP	0.94	0.88	0.88	0.93	3.3	0.53			0.22*	0.006
CP	0.96	0.91	*Nc*.	0.95	2.9	0.72			0.07	0.413
AC^**b**^	0.82	0.67	0.63	0.84	2.9	0.72			0.30*	<0.001
Total^c^	0.94	0.82	0.62	0.97	3.2	0.39				

Note

α Cronbach’s alpha

β Revelle’s beta

λ_6_ Guttman’s lambda-6

ω_h_ McDonald’s omega hierarchical. *Nc*. = Not calculated

*r* Pearson’s correlation coefficient.

*significant correlation at *p* = 0.01

^a^ Intention without INT1

^b^ Action control without AC2

^c^ Total = remaining set of 32 items

The corrected item total correlations for the remaining set of items ranged from 0.24 to 0.78, with one item below threshold, namely OE1 (0.24). The corrected item total correlations ranged from 0.38 (OE1) to 0.95 (CP1) for the subscales. This indicated that each item correlated and the its scale score without that item. The reliability measures of the subscales increased when item OE1 was removed from the subscale outcome expectancies and item INT4 was removed from the three-item intention subscale. Because a minimum of three items per factor are needed [34] and a subscale of three items is preferred over a subscale of two items for a reliable subscale assessment [35], it was decided to maintain both OE1 and INT4. The reliability coefficients confirmed the decision to remove INT1 and AC2 from their subscales.

#### Step 6. Total (sub)scale scores

The mean scores of the subscales ranged from 2.7 for the action control to 3.5 for coping self-efficacy. Pearson correlations coefficients between the subscales ranged from 0.09 to 0.65 (Table 6).

**Table 6:** Pearson correlation coefficients between the HAPA subscales (pairwise deletion).

	outcome expectancies	risk perceptions	action self-efficacy	intention	coping self-efficacy	action planning	coping planning
risk perception	0.15						
action self-efficacy	0.16	0.28*					
Intention	0.19	0.21*	0.47*				
coping self-efficacy	0.16	0.30*	0.60*	0.45*			
action planning	0.19	0.44*	0.47*	0.35*	0.65*		
coping planning	0.24*	0.34*	0.35*	0.36*	0.53*	0.65*	
action control	0.14	0.09	0.20	0.25*	0.34*	0.38*	0.44*

* *p*-values <0.010 (2-tailed)

^d^ Variations in total number of cases are the results of case-by-case analysis. Therefore, the minimum number of cases for each subgroup is indicated.

### PCA

The principal component analysis with the entire set of 34 items and varimax rotation and Eigenvalue > 1 extracted nine components, which explained 79.6% of the variance in the items (*KMO* = 0.849). The action control items were divided into two components. One item [INT4] had factor loadings of < 0.5 in its anticipated component and a factor loading of > 0.5 on another component [action control]. Based on the results of the Dima protocol analyses, we also performed a PCA of the remaining set of items [without INT1 and AC2], which extracted eight components and explained 78.9% of the variance *(KMO* = 0.849) (S2 Fig). The outcome of the PCA verified the results of the Dima protocol analyses.

### Convergent and discriminant validity

The HAPA subscales, except outcome expectancies, correlated significantly. There were weak but significant correlations between daily brushing frequency (open-ended question) and the subscales action planning and action control (Table 5).

## Discussion

The aim of this study was to improve the existing HAPA-based questionnaire for parents regarding brushing their children’s teeth in a population of parents of high caries risk children by identifying the individual constructs and to assess its validity and reliability. The improved HAPA-based questionnaire had a multidimensional scale. After the removal of two of the 34 items, the eight HAPA constructs could be identified as separate subscales. With the exception of action control, these eight HAPA constructs represented unidimensional scales with good homogeneity. Exploratory factor analysis identified eight factors. However, the confirmatory factor analysis indicated that a nine-factor model (with two factors for the action control subscale) had the best fit for the data. The lack of fit of the graded response model indicated that the model was too restrictive for our data. All the subscales had excellent reliability.

However, it also appeared that the items in the subscales were highly correlated and showed small variance. It might be that the content of the items within the subscales was too similar. Our data suggested some adjustments of the questionnaire. The first suggestion is to exclude one item of the subscale intention (‘*In the period ahead*, *I intend to…*’). It is to be expected that parents who brushed more than twice a day probably agreed with the item:’*… brush my child’s teeth properly at least twice a day*’ and disagreed with item: ‘…*brush my child’s teeth properly once a day’*. This could result in a lower score on the intention subscale than could be expected based on their latent trait. We decided to remove the latter item (‘*In the period ahead*, *I intend to brush my child’s teeth properly once a day’*) and maintain the former item, since that item is more indicative of an optimal intention than the latter item. Secondly, removal of an item of the subscale action control is suggested *(*‘*In the past week I knew exactly when I skipped brushing my child’s teeth’)*. This item was not scalable and therefore removed. The problem might be that this item measured awareness of a non-performance, while the other action control items focused on the performance of a task. Thirdly, factor analysis revealed that the subscale action control consisted of a two-factor structure, namely self-monitoring and awareness of standards (e.g., memorising the goals). Since action control consists of awareness of standards, self-monitoring and self-regulation [3, 36], we decided to maintain one action control subscale.

The reliability of the questionnaire was satisfactory. The reliability measures suggested removal of one item from the outcome expectancies subscale. However, as noted above, we decided to maintain this item to preserve the broad spectrum of the construct.

The convergent validity was good, since the HAPA subscales correlated significantly. The discriminant validity of the questionnaire was sufficient. As expected, higher brushing frequency was related to higher mean scores on the volitional constructs action planning and action control.

The results of the study must be interpreted in the context of its limitations. To start with, this study did not include test-retest stability. However, since our previous study showed excellent reproducibility (17), it seemed unlikely that this would have declined after our adaptations of the questionnaire. Furthermore, the item order might have influenced the outcomes. In the preceding study, the items were inter-mixed, while in the current study the items were grouped per anticipated construct using section breaks and highlighting item stems. Chan et al. [37] previously assessed the effect of item order in questionnaires. The results indicated that ensemble-order (items of one construct are presented subsequentially), especially when an item stem is highlighted and section breaks are placed, as we did in our present study, produced less bias (random measurement error) than inter-mixing the items [37]. Our sample size was relatively small, and we did not conduct power analyses for the determination of sample size. It is theoretically possible that fewer factors may have been identified than if we had a larger sample. However, based on our results, we do not anticipate any added value from a larger sample. On the contrary, employing a larger sample would impose a greater burden on respondents, extend the duration of the research, and result in higher costs.

In conclusion, the applied revisions improved the questionnaire substantially. We intended to create a multidimensional questionnaire with eight HAPA constructs and we succeeded. The findings of this validation study suggest that, after the removal of two items, the adapted questionnaire is a valid and reliable instrument to evaluate HAPA-based interventions to improve children’s oral health. Future researchers could analyse the HAPA model in a sample with more variation in caries risk by looking into the pathways between the social cognitive constructs of the HAPA model.

## Supporting information

S1 Checklist Human participants research checklist. (DOCX)

S1 Fig The HAPA model, adapted from Schwarzer. (TIF)

S2 Fig Scree plot principal component analyses with the remaining set of 32 HAPA items [without INT1 and AC2], with varimax rotation and Eigenvalue >1. (TIF)

S1 Table Description of the HAPA social cognitive constructs for the motivational and volitional phases. (DOCX)

S2 Table Pearson’s inter-item correlations coefficients after missing value imputation (*n* = 160). (DOCX)
