# Supplementary figures and images for: Validation of an improved questionnaire assessing the social cognitive constructs of the Health Action Process Approach among parents regarding brushing their children’s teeth
Source: PLoS One. 2024 Jun 4;19(6):e0300432. doi: 10.1371/journal.pone.0300432 (PMC11149846; doi:10.1371/journal.pone.0300432)

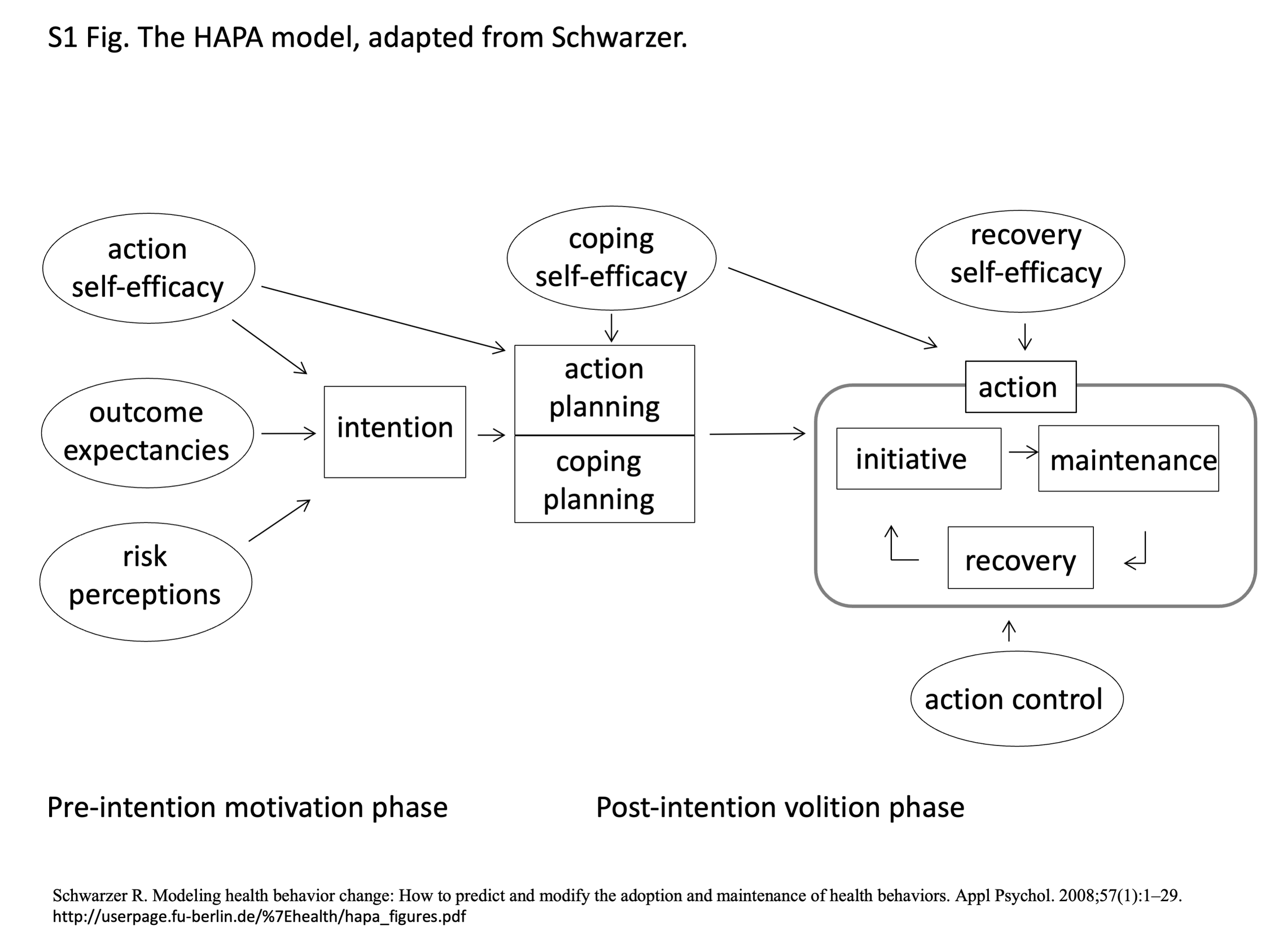

Supplement: S1 Fig — (TIF) [file pone.0300432.s002.tif]

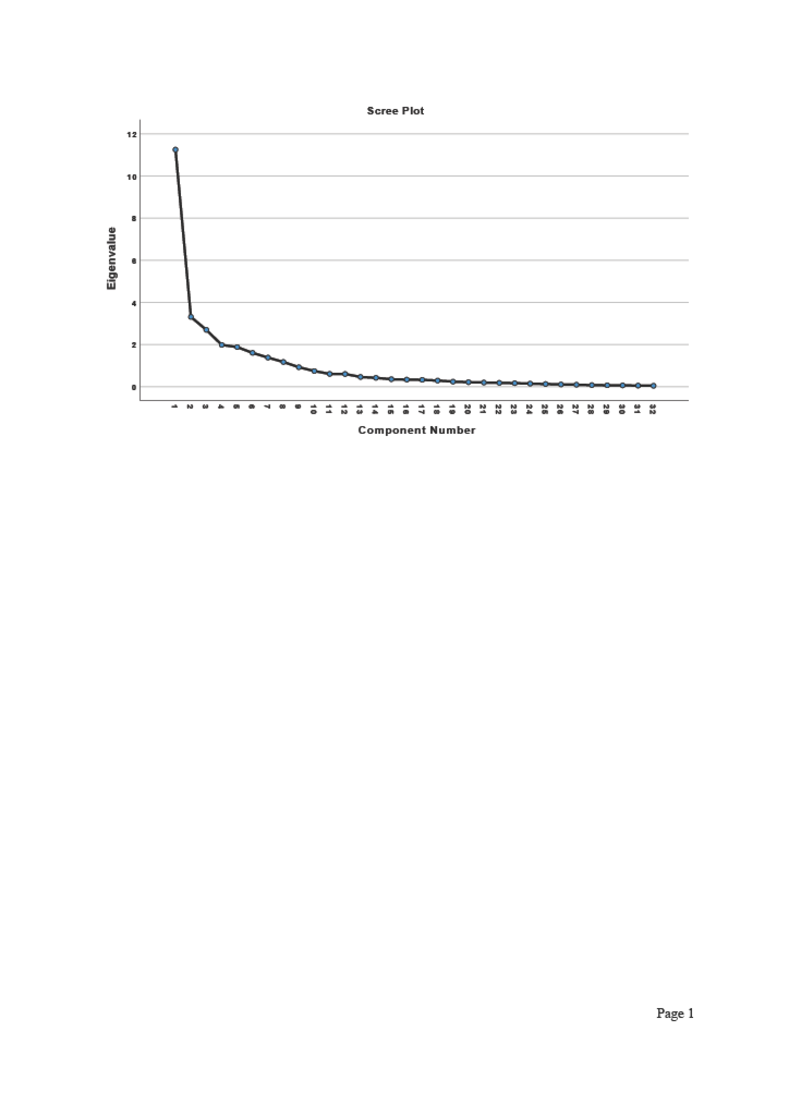

Supplement: S2 Fig — (TIF) [file pone.0300432.s003.tif]
