# Supplementary material for: Validation of an improved questionnaire assessing the social cognitive constructs of the Health Action Process Approach among parents regarding brushing their children’s teeth
Source: PLoS One. 2024 Jun 4;19(6):e0300432. doi: 10.1371/journal.pone.0300432 (PMC11149846; doi:10.1371/journal.pone.0300432)
Supplement: S1 Table — (DOCX) [file pone.0300432.s004.docx]

| **Construct** | **Abbr.** | **Description** |
| --- | --- | --- |
| Motivational phase |  |  |
| Outcome expectancies | OE | Understanding of the contingencies between a person’s actions and subsequent outcomes |
| Risk perceptions | RP | Perceived severity of a health condition and personal vulnerability toward it |
| Action self-efficacy | aSE | Beliefs in one’s capabilities to exercise control over challenging demands and over one’s own functioning |
| Intention | INT | Motivation to alter the previous way of life and set goals for a different course of action |
| Volitional phase |  |  |
| Coping self-efficacy | cSE | Optimistic beliefs about one’s capability to cope with barriers that arise during the period of behavioural maintenance |
| Action planning | AP | Planning to connecting the individual with good opportunities to act trough a task-facilitation strategy |
| Coping planning | CP | Protecting good intention from anticipated obstacles via a distraction-inhibiting strategy |
| Action control | AC | Self-regulatory strategy for promoting maintenance of an enacted behaviour through the continual monitoring and evaluation of a behaviour against a desired behavioural standard. |

S1_Table. Description of the HAPA social cognitive constructs for the motivational and volitional phases.
