# Supplementary material for: Validation of an improved questionnaire assessing the social cognitive constructs of the Health Action Process Approach among parents regarding brushing their children’s teeth
Source: PLoS One. 2024 Jun 4;19(6):e0300432. doi: 10.1371/journal.pone.0300432 (PMC11149846; doi:10.1371/journal.pone.0300432)
Supplement: S2 Table — (DOCX) [file pone.0300432.s005.docx]

Supplementary Table 2 Pearson’s inter-item correlations coefficients after missing value imputation ($n=$160).

|  | INT1 | INT2 | INT3 | INT4 | OE1 | OE2 | OE3 | RP1 | RP2 | RP3 | RP4 | RP5 | aSE1 | aSE2 | aSE3 | cSE1 | cSE2 | cSE3 | cSE4 | AP1 | AP2 | AP3 | AP4 | AP5 | CP1 | CP2 | CP3 | CP4 | AC1 | AC2 | AC3 | AC4 | AC5 |
| --- | --- | --- | --- | --- | --- | --- | --- | --- | --- | --- | --- | --- | --- | --- | --- | --- | --- | --- | --- | --- | --- | --- | --- | --- | --- | --- | --- | --- | --- | --- | --- | --- | --- |
| INT2 | 0.42^a^ |  |  |  |  |  |  |  |  |  |  |  |  |  |  |  |  |  |  |  |  |  |  |  |  |  |  |  |  |  |  |  |  |
| INT3 | 0.35^a^ | 0.61^a^ |  |  |  |  |  |  |  |  |  |  |  |  |  |  |  |  |  |  |  |  |  |  |  |  |  |  |  |  |  |  |  |
| INT4 | 0.12 | 0.33^a^ | 0.50^a^ |  |  |  |  |  |  |  |  |  |  |  |  |  |  |  |  |  |  |  |  |  |  |  |  |  |  |  |  |  |  |
| OE1 | 0.15 | 0.12 | 0.09 | 0.16 |  |  |  |  |  |  |  |  |  |  |  |  |  |  |  |  |  |  |  |  |  |  |  |  |  |  |  |  |  |
| OE2 | 0.12 | 0.25^a^ | 0.22^a^ | 0.24^a^ | 0.34^a^ |  |  |  |  |  |  |  |  |  |  |  |  |  |  |  |  |  |  |  |  |  |  |  |  |  |  |  |  |
| OE3 | 0.12 | 0.11 | 0.08 | 0.13 | 0.35^a^ | 0.73^a^ |  |  |  |  |  |  |  |  |  |  |  |  |  |  |  |  |  |  |  |  |  |  |  |  |  |  |  |
| RP1 | 0.08 | 0.17^b^ | 0.16^b^ | 0.08 | **-0.01** | 0.18^b^ | 0.22^a^ |  |  |  |  |  |  |  |  |  |  |  |  |  |  |  |  |  |  |  |  |  |  |  |  |  |  |
| RP2 | 0.12 | 0.27^a^ | 0.18^b^ | 0.16^b^ | 0.01 | 0.26^a^ | 0.27^a^ | 0.69^a^ |  |  |  |  |  |  |  |  |  |  |  |  |  |  |  |  |  |  |  |  |  |  |  |  |  |
| RP3 | 0.03 | 0.20^b^ | 0.17^b^ | 0.13 | **-0.08** | 0.20^a^ | 0.17^b^ | 0.66^a^ | 0.79^a^ |  |  |  |  |  |  |  |  |  |  |  |  |  |  |  |  |  |  |  |  |  |  |  |  |
| RP4 | 0.05 | 0.15 | 0.10 | 0.09 | 0.01 | 0.23^a^ | 0.23^a^ | 0.57^a^ | 0.70^a^ | 0.77^a^ |  |  |  |  |  |  |  |  |  |  |  |  |  |  |  |  |  |  |  |  |  |  |  |
| RP5 | 0.11 | 0.18^b^ | 0.11 | 0.09 | **-0.01** | 0.17^b^ | 0.18^b^ | 0.44^a^ | 0.56^a^ | 0.58^a^ | 0.61^a^ |  |  |  |  |  |  |  |  |  |  |  |  |  |  |  |  |  |  |  |  |  |  |
| aSE1 | 0.06 | 0.39^a^ | 0.40^a^ | 0.30 ^a^ | 0.07 | 0.25^a^ | 0.12 | 0.32^a^ | 0.24^a^ | 0.24^a^ | 0.21^a^ | 0.15 |  |  |  |  |  |  |  |  |  |  |  |  |  |  |  |  |  |  |  |  |  |
| aSE2 | 0.11 | 0.41^a^ | 0.41^a^ | 0.31 ^a^ | 0.08 | 0.27^a^ | 0.15 | 0.28^a^ | 0.24^a^ | 0.21^a^ | 0.22^a^ | 0.19^b^ | 0.93^a^ |  |  |  |  |  |  |  |  |  |  |  |  |  |  |  |  |  |  |  |  |
| aSE3 | 0.06 | 0.37^a^ | 0.36^a^ | 0.26 ^a^ | 0.10 | 0.23^a^ | 0.08 | 0.23^a^ | 0.22^a^ | 0.22^a^ | 0.26^a^ | 0.21^a^ | 0.82^a^ | 0.88^a^ |  |  |  |  |  |  |  |  |  |  |  |  |  |  |  |  |  |  |  |
| cSE1 | 0.18^b^ | 0.35^a^ | 0.47^a^ | 0.47 ^a^ | 0.10 | 0.26^a^ | 0.12 | 0.23^a^ | 0.31^a^ | 0.19^b^ | 0.20^a^ | 0.23^a^ | 0.58^a^ | 0.64^a^ | 0.62^a^ |  |  |  |  |  |  |  |  |  |  |  |  |  |  |  |  |  |  |
| cSE2 | 0.17^b^ | 0.33^a^ | 0.42^a^ | 0.41 ^a^ | 0.12 | 0.19^b^ | 0.09 | 0.22^a^ | 0.29^a^ | 0.19^b^ | 0.23^a^ | 0.26^a^ | 0.50^a^ | 0.57^a^ | 0.57^a^ | 0.90^a^ |  |  |  |  |  |  |  |  |  |  |  |  |  |  |  |  |  |
| cSE3 | 0.08 | 0.27^a^ | 0.34^a^ | 0.37 ^a^ | 0.14 | 0.18^b^ | 0.16^b^ | 0.24^a^ | 0.25^a^ | 0.18^b^ | 0.12 | 0.27^a^ | 0.48^a^ | 0.55^a^ | 0.54^a^ | 0.81^a^ | 0.83^a^ |  |  |  |  |  |  |  |  |  |  |  |  |  |  |  |  |
| cSE4 | 0.10 | 0.31^a^ | 0.39^a^ | 0.41 ^a^ | 0.10 | 0.28^a^ | 0.14 | 0.21^a^ | 0.27^a^ | 0.17^b^ | 0.15 | 0.23^a^ | 0.52^a^ | 0.59^a^ | 0.59^a^ | 0.89^a^ | 0.84^a^ | 0.83^a^ |  |  |  |  |  |  |  |  |  |  |  |  |  |  |  |
| AP1 | 0.16^b^ | 0.18^b^ | 0.22^a^ | 0.30 ^a^ | 0.19^b^ | 0.22^a^ | 0.25^a^ | 0.36^a^ | 0.33^a^ | 0.30^a^ | 0.32^a^ | 0.19^b^ | 0.43^a^ | 0.43^a^ | 0.45^a^ | 0.52^a^ | 0.52^a^ | 0.47^a^ | 0.51^a^ |  |  |  |  |  |  |  |  |  |  |  |  |  |  |
| AP2 | 0.12 | 0.25^a^ | 0.29^a^ | 0.41 ^a^ | 0.15 | 0.27^a^ | 0.22^a^ | 0.37^a^ | 0.34^a^ | 0.30^a^ | 0.25^a^ | 0.22^a^ | 0.49^a^ | 0.47^a^ | 0.45^a^ | 0.59^a^ | 0.54^a^ | 0.51^a^ | 0.54^a^ | 0.76^a^ |  |  |  |  |  |  |  |  |  |  |  |  |  |
| AP3 | 0.06 | 0.22^a^ | 0.31^a^ | 0.44^a^ | 0.10 | 0.27^a^ | 0.25^a^ | 0.28^a^ | 0.31^a^ | 0.28^a^ | 0.31^a^ | 0.25^a^ | 0.44^a^ | 0.48^a^ | 0.49^a^ | 0.62^a^ | 0.61^a^ | 0.55^a^ | 0.62^a^ | 0.70^a^ | 0.82^a^ |  |  |  |  |  |  |  |  |  |  |  |  |
| AP4 | 0.14 | 0.23^a^ | 0.29^a^ | 0.38^a^ | 0.13 | 0.21^a^ | 0.19^b^ | 0.30^a^ | 0.34^a^ | 0.29^a^ | 0.30^a^ | 0.32^a^ | 0.36^a^ | 0.40^a^ | 0.42^a^ | 0.58^a^ | 0.62^a^ | 0.57^a^ | 0.60^a^ | 0.71^a^ | 0.76^a^ | 0.81^a^ |  |  |  |  |  |  |  |  |  |  |  |
| AP5 | 0.07 | 0.22^a^ | 0.31^a^ | 0.37^a^ | 0.12 | 0.23^a^ | 0.13 | 0.37^a^ | 0.36^a^ | 0.33^a^ | 0.30^a^ | 0.30^a^ | 0.42^a^ | 0.43^a^ | 0.40^a^ | 0.58^a^ | 0.59^a^ | 0.56^a^ | 0.60^a^ | 0.66^a^ | 0.73^a^ | 0.79^a^ | 0.84^a^ |  |  |  |  |  |  |  |  |  |  |
| CP1 | 0.18^b^ | 0.22^a^ | 0.34^a^ | 0.33^a^ | 0.21^a^ | 0.22^a^ | 0.20^b^ | 0.31^a^ | 0.26^a^ | 0.27^a^ | 0.28^a^ | 0.22^a^ | 0.29^a^ | 0.34^a^ | 0.32^a^ | 0.46^a^ | 0.49^a^ | 0.43^a^ | 0.47^a^ | 0.57^a^ | 0.52^a^ | 0.52^a^ | 0.57^a^ | 0.59^a^ |  |  |  |  |  |  |  |  |  |
| CP2 | 0.22^a^ | 0.22^a^ | 0.33^a^ | 0.34^a^ | 0.22^a^ | 0.24^a^ | 0.20^b^ | 0.28^a^ | 0.24^a^ | 0.24^a^ | 0.28^a^ | 0.24^a^ | 0.32^a^ | 0.38^a^ | 0.36^a^ | 0.48^a^ | 0.53^a^ | 0.50^a^ | 0.48^a^ | 0.59^a^ | 0.53^a^ | 0.54^a^ | 0.61^a^ | 0.60^a^ | 0.91^a^ |  |  |  |  |  |  |  |  |
| CP3 | 0.18^b^ | 0.27^a^ | 0.35^a^ | 0.31^a^ | 0.17^b^ | 0.25^a^ | 0.20^b^ | 0.30^a^ | 0.28^a^ | 0.32^a^ | 0.32^a^ | 0.30^a^ | 0.35^a^ | 0.39^a^ | 0.38^a^ | 0.48^a^ | 0.51^a^ | 0.49^a^ | 0.45^a^ | 0.53^a^ | 0.51^a^ | 0.51^a^ | 0.54^a^ | 0.57^a^ | 0.87^a^ | 0.88^a^ |  |  |  |  |  |  |  |
| CP4 | 0.18^b^ | 0.24^a^ | 0.36^a^ | 0.33^a^ | 0.20^b^ | 0.16^b^ | 0.13 | 0.30^a^ | 0.20^b^ | 0.24^a^ | 0.27^a^ | 0.27^a^ | 0.31^a^ | 0.34^a^ | 0.36^a^ | 0.48^a^ | 0.51^a^ | 0.52^a^ | 0.48^a^ | 0.55^a^ | 0.50^a^ | 0.52^a^ | 0.59^a^ | 0.63^a^ | 0.80^a^ | 0.81^a^ | 0.79^a^ |  |  |  |  |  |  |
| AC1 | 0.06 | 0.08 | 0.12 | 0.26^a^ | 0.20^b^ | **-0.01** | 0.02 | 0.13 | 0.11 | 0.11 | 0.06 | 0.09 | 0.13 | 0.13 | 0.09 | 0.32^a^ | 0.33^a^ | 0.31^a^ | 0.32^a^ | 0.33^a^ | 0.40^a^ | 0.37^a^ | 0.45^a^ | 0.37^a^ | 0.37^a^ | 0.39^a^ | 0.35^a^ | 0.39^a^ |  |  |  |  |  |
| AC2 | 0.12 | 0.26^a^ | 0.29^a^ | 0.32 | 0.01 | **-0.01** | **-0.10** | 0.08 | 0.07 | 0.08 | 0.07 | **-0.03** | 0.29^a^ | 0.28^a^ | 0.32^a^ | 0.29^a^ | 0.28^a^ | 0.21^a^ | 0.24^a^ | 0.20^b^ | 0.28^a^ | 0.31^a^ | 0.21^a^ | 0.22^a^ | 0.31^a^ | 0.32^a^ | 0.36^a^ | 0.30^a^ | 0.28^a^ |  |  |  |  |
| AC3 | 0.10 | 0.02 | 0.17^b^ | 0.12 | 0.16^b^ | 0.01 | 0.10 | 0.04 | 0.04 | 0.00 | 0.07 | 0.04 | 0.05 | 0.07 | 0.07 | 0.12 | 0.12 | 0.07 | 0.12 | 0.16 | 0.16^b^ | 0.19^b^ | 0.21^a^ | 0.18^b^ | 0.31^a^ | 0.30^a^ | 0.27^a^ | 0.32^a^ | 0.48^a^ | 0.26^a^ |  |  |  |
| AC4 | 0.14 | 0.10 | 0.17^b^ | 0.15 | 0.21^a^ | 0.06 | 0.08 | 0.04 | 0.02 | -0.02 | 0.03 | 0.00 | 0.06 | 0.06 | 0.12 | 0.19^b^ | 0.24 ^a^ | 0.17^b^ | 0.22^a^ | 0.20^b^ | 0.14 | 0.15 | 0.25^a^ | 0.13 | 0.33^a^ | 0.31^a^ | 0.27^a^ | 0.30^a^ | 0.52^a^ | 0.28^a^ | 0.*^67a^* |  |  |
| AC5 | **-0.08** | 0.18^b^ | 0.21^a^ | 0.36^a^ | 0.26^a^ | 0.14 | 0.06 | 0.12 | 0.15 | 0.09 | 0.12 | 0.17^b^ | 0.28^a^ | 0.29^a^ | 0.33^a^ | 0.38^a^ | 0.37 ^a^ | 0.39^a^ | 0.41^a^ | 0.33^a^ | 0.42^a^ | 0.46^a^ | 0.45^a^ | 0.45^a^ | 0.35^a^ | 0.39^a^ | 0.32^a^ | 0.38^a^ | 0.50^a^ | 0.33^a^ | 0.32^a^ | 0.43^a^ |  |
| AC6 | 0.04 | 0.22^a^ | 0.28^a^ | 0.35^a^ | 0.16^b^ | 0.07 | 0.02 | 0.11 | 0.18^b^ | 0.13 | 0.10 | 0.11 | 0.28^a^ | 0.29^a^ | 0.33^a^ | 0.41^a^ | 0.41^a^ | 0.39^a^ | 0.40^a^ | 0.28^a^ | 0.35^a^ | 0.40^a^ | 0.40^a^ | 0.36^a^ | 0.33^a^ | 0.38^a^ | 0.34^a^ | 0.38^a^ | 0.44^a^ | 0.39^a^ | 0.36^a^ | 0.44^a^ | 0.76^a^ |

Negative correlations are in bold

^a^ Correlation is significant at the 0.01 level (2-tailed)

^b^ Correlation is significant at the 0.05 level (2-tailed)
